# CD160 receptor in CLL: Current state and future avenues

**DOI:** 10.3389/fimmu.2022.1028013

**Published:** 2022-11-07

**Authors:** Loubna Oumeslakht, Abdel-ilah Aziz, Armand Bensussan, Sanae Ben Mkaddem

**Affiliations:** ^1^ Institute of Biological Sciences, Mohammed VI Polytechnic University, Ben-Guerir, Morocco; ^2^ INSERM U976, Université de Paris, Hôpital Saint Louis, Paris, France; ^3^ Institut Jean Godinot, Centre de Lutte Contre le Cancer, Reims, France

**Keywords:** chronic lymphocytic leukemia, CD160, NK cells, minimal residual disease, prognosis

## Abstract

CD160 is a glycosylphosphatidylinositol (GPI)-anchored cell surface glycoprotein expressed on cytotoxic natural killer (NK) cells and T-cell subsets. It plays a crucial role in the activation of NK-cell cytotoxicity and cytokine production. It also modulates the immune system and is involved in some pathologies, such as cancer. CD160 is abnormally expressed in B-cell chronic lymphocytic leukemia (CLL) but not expressed in normal B lymphocytes. Its expression in CLL enhances tumor cell proliferation and resistance to apoptosis. CD160 is also a potential prognostic marker for the detection of minimal residual disease (MRD) in CLL, which is important for the clinical management of CLL, the prevention of disease relapse, and the achievement of complete remission. In this review, we present an overview of CD160 and its involvement in the pathophysiology of CLL. We also discuss its use as a prognostic marker for the assessment of MRD in CLL.

## 1 Introduction

CD160 is a glycophosphatidylinositol (GPI)-anchored surface membrane protein that was initially identified as BY55 by Maïza et al. on cytotoxic natural killer (NK) cells, where it activates NK-cell cytotoxicity and cytokine production (1–3). CD160 is also expressed by some subsets of T cells and activated endothelial cells, in which it regulates cell activation and apoptosis, respectively (4–7). Thus, CD160 is involved in antitumor immunity (3) and protection during chronic infections (8, 9). In general, CD160 has been reported to be involved in the development of some pathologies, including autoimmune diseases (10), inflammatory diseases (11), atherosclerosis (12), retinal vascular diseases (7), chronic viral infections (8, 13–15), and cancer (16–18).

CD160 is abnormally overexpressed in B-chronic lymphocytic leukemia (CLL), which is the most frequently diagnosed leukemia in developed countries (19, 20). The disease is characterized by the clonal expansion and accumulation of small mature-like CD5^+^ CD23^+^ B cells in the blood, bone marrow, and secondary lymphoid tissues (21). Different factors have been implicated in CLL progression. First, the genetic and epigenetic profiles of CLL B cells are altered (22, 23). Second, CLL B cells interact with neighboring cells in their microenvironment, such as nurse-like cells (24–28), endothelial cells (29–31), mesenchymal stromal cells (32), and T cells (33–35). These cells play a crucial role in the maintenance of CLL B-cell proliferation and CLL progression, the enhancement of prosurvival signaling, and the induction of resistance to drug-induced apoptosis (36). The microenvironment is also characterized by exhausted cytotoxic T cells and NK cells, allowing CLL B cells to escape the immune system (33, 37–39). Third, B-cell receptor (BCR) signaling plays a key pathological role by activating the signaling pathways implicated in CLL cell survival, metabolism, proliferation, and resistance to apoptosis (40–42). Furthermore, CLL is characterized by the expression of several markers specific to certain immune cells, such as ζ-associated protein kinase 70 (ZAP-70) (43) and lymphocyte-specific tyrosine kinase (Lck) (44), which favor CLL cell survival, and CD5, which maintains CLL cell anergy (45). CD160 is also a crucial marker of CLL and a key activator of CLL cell survival and resistance to apoptosis (46).

Nonetheless, CLL remains an incurable disease. It can be treated by chemoimmunotherapy (CIT) using a combination of fludarabine, cyclophosphamide, and rituximab (FCR) or chlorambucil and obinutuzumab (CLBO) (47) or by targeted therapy using small-molecule inhibitors targeting various mediators involved in BCR signaling, such as BTK inhibitors (BTKis) (ibrutinib and acalabrutinib) (48), PI3K inhibitors (PI3Kis) (idelalisib and duvelisib) (49), and Bcl-2 (B-cell lymphoma 2) antagonists (venetoclax) (50). These molecules can be prescribed as frontline or second-line treatments depending on the existence/type of IGHV and p53 mutations (47). They have improved the prognosis of patients for whom CIT is not suited (47). However, CLL patients can develop resistance to these treatments due to secondary mutations in the proteins targeted by the drugs and the development of alternative bypass pathways (51). In addition, resistance can be caused by the activation of survival and antiapoptotic signaling pathways triggered through crosstalk between CLL components and surrounding cells in the microenvironment (51). Though these treatments can improve survival rates, some of them have adverse effects (52, 53). Therefore, for clinical management of this disease, it is important to establish a good follow-up strategy to analyze the development or regression of CLL in patients after treatment. Hence, the assessment of minimal residual disease remains crucial for the achievement of complete remission, relapse prevention, and prolonged survival.

The restricted expression of CD160 in CLL and lack of CD160 expression in normal B cells make it a good potential diagnostic marker but also a prognostic marker for the detection of minimal residual disease in CLL (54). In this review, we discuss the characteristics of CD160, its expression in the CLL tumor microenvironment, and its roles in the pathophysiological processes of CLL. We also report the use of CD160 as a potential prognostic marker for the assessment of minimal residual disease in CLL.

## 2 General characteristics of CD160

**Figure 1:**
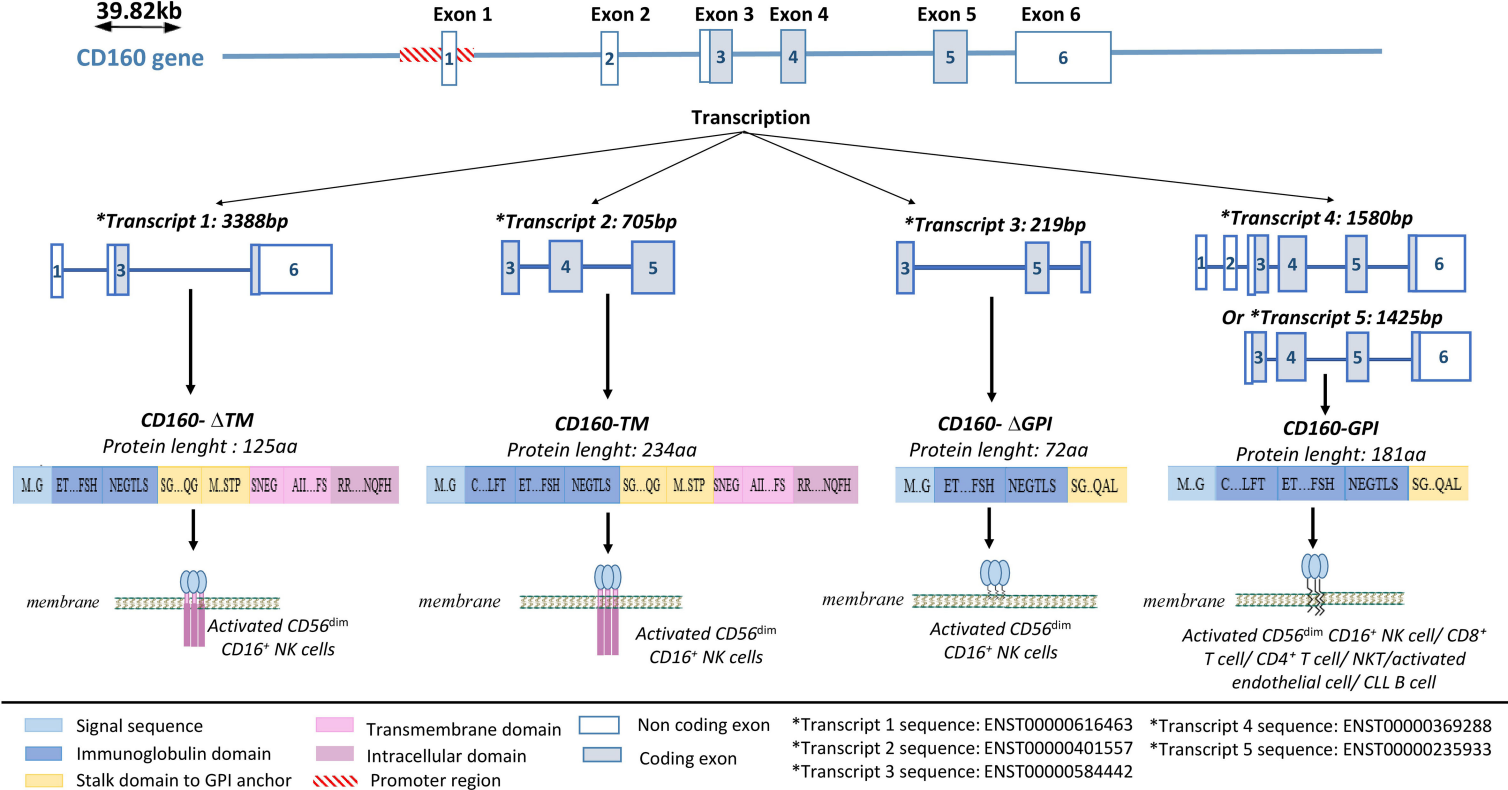
CD160 gene structure and isoforms. CD160 has four isoforms: a transmembrane isoform (CD160-TM), a glycophosphatidylinositol-anchored isoform (CD160-GPI), and two truncated isoforms (CD160-ΔIg-GPI and CD160-ΔIg-TM) that are generated through alternative splicing. Adapted from (57) and analyzed on Ensembl.

CD160 is a receptor that is capable of delivering both stimulatory and inhibitory signals depending on the type of cell in which it is expressed (4, 55). Its gene is located on the human 1q21.1 chromosome. It contains 6 exons, with exons 1 and 2 being untranslated (56) (**Figure 1**). A core promoter sequence containing a single transcription start site (TSS) is located upstream of exon 1 (56). This sequence is homologous to three highly conserved transcription factor-binding sites for FREAC-4, SOX17, and acute myelogenous leukemia-1 (AML-1) (also called RUNX1 or CBFα). The AML-1 binding site is the only one that has been implicated in the regulation of CD160 expression (56). However, data explaining the functional properties of this binding site and the mechanism by which CD160 expression is controlled in immune cells are needed.

### 2.1. CD160 isoforms

Four CD160 isoforms are generated by alternative splicing, and they differ based on the presence or absence of a GPI anchor, an Ig domain, and transmembrane and cytoplasmic domains (57); a transmembrane form (CD160-TM), two truncated forms (CD160-ΔIg-GPI and CD160-ΔIg-TM), a GPI-anchored membrane form (CD160-GPI), and a soluble form (sCD160) (57, 58) (**Figure 1**).

CD160-GPI has a single Ig-like domain that is weakly homologous to the KIR2DL4 receptor and is expressed in peripheral blood (PB) CD56^dim^ CD16^+^ NK-cell subsets (59, 60), cytotoxic CD8^+^ T cells (59), activated endothelial cells (7, 61), a small fraction of CD4^+^ T cells (4) and γδ-T cells (1). CD160-TM is selectively expressed by activated CD56^dim^ CD16^+^ NK cells (57). Its expression is activation-dependent and amplifies NK-cell cytotoxicity (57). CD4^+^ and CD8^+^ T cells can also express CD160-TM, but only at the transcriptional level and not at the protein level (5). Soluble CD160 can be secreted by human PB-NK cells (58), murine splenic CD8^+^ T cells (62), and human and murine mast cells (63). In some pathological conditions, sCD160 can also be expressed by tumor cells, such as melanoma cells (18) (**Table 1**).

**Table 1:** Cell expression and functions of CD160 isoforms.

CD160 isoform	Expression	Function	References
**CD160-GPI**	CD56^dim^ CD16^+^ NK cells	Activation of NK cell cytotoxicity and metabolism	(3, 14, 55, 59, 60, 64)
	CD8^+^ T cells	Activation or inhibition of CD8^+^ T cell activity	(6, 8, 9, 15, 65)
	Activated endothelial cells	Induction of cell apoptosis	(7, 17, 61, 66)
	CD4^+^ T cells	Activation or inhibition of CD4^+^ T cell proliferation and cytokine production	(4, 5, 67–71)
	NKT cells	Inhibition of cell functions	(72)
	Malignant B cells in chronic lymphocytic leukaemia	Activation of cell proliferation and Antiapoptosis	(19, 46)
**CD160-TM**	Activated CD56^dim^ CD16^+^ NK cells	Amplification of cell cytotoxicity	(57)
**sCD160**	Activated CD56^dim^ CD16^+^ NK cells	Immunoregulation and inhibition of CD8^+^ T cell cytotoxicity	(58)
	Activated murine CD8^+^ T cells	Immunoregulation	(62)
	mast cells	Immunoregulation and inhibition of CD8^+^ T cells cytotoxicity	(63)
	Melanoma cells	Inhibition of NK-cells cytotoxicity	(18)
**Truncated CD160 isoforms (CD160-ΔIg-GPI and CD160-ΔIg-TM),**	CD56^dim^ CD16^+^ NK cells	Unknown function	(57)

CD160-GPI: CD160-glycophosphatidyl inositol; CD160-TM: CD160-transmembrane; sCD160: soluble CD160.

Both human and murine CD160 exhibit a broad but weak specificity for classical and nonclassical MHC-I molecules (73). For instance, human CD160 preferentially binds to HLA-C (3) and soluble HLA-G (61). CD160 can also bind to HLA-A2 tetramers, HLA-E, and HLA-B7 (74). In addition, CD160 binds to herpesvirus entry mediator (HVEM), which is in the tumor necrosis factor (TNF) superfamily (75). The CD160-TM isoform binds less robustly to HVEM (5).

### 2.2. Physiological and pathological functions of CD160

#### 2. 2. 1 CD160 function in NK cells

CD160 plays a major role in the induction of CD56^dim^ CD16^+^ NK-cell cytotoxicity and degranulation (60) (**Table 1**). However, it is not expressed on CD56^bright^ CD16^⁻^ NK cells, which exert immunoregulatory functions through the expression of high levels of cytokines and chemokines (60). Upon engagement with MHC-I molecules, HVEM or agonistic monoclonal anti-CD160 antibodies, including BY55 and CL1-R2, on PB-CD56^dim^ CD16^+^ NK cells, CD160-GPI activates cytotoxic functions and enhances the production of cytokines, including interferon-γ (IFN-γ), TNF-α, interleukin-6 (IL-6), IL-8 and MIP-1b, which induce target cell death (3, 60, 64). The CD160 cytotoxic effect does not depend on cross-linking with other activating NK-cell receptors, such as activating killer Ig-like receptors (KIRs) or CD94/NK2C, and its effect is similar to that mediated by CD16 (60). In addition, the expression of the CD160-TM isoform by activated NK cells can exacerbate toxicity (57). CD160 mediates cytotoxicity through the recruitment of spleen-associated tyrosine kinase (Syk) (55), which activates the PI3K/Akt/mTORC1 signaling pathway, promoting translation through the upregulation of 4EBP1 and S6 ribosomal kinase expression (55, 76). Furthermore, the PI3K/Akt pathway can also activate NK-cell migration through the phosphorylation of MEK1/2-ERK, which has been implicated in actin reorganization and cell polarization (55, 77). CD160-GPI does not contain immunoreceptor tyrosine-based motifs (ITAMs), and it is not yet clear how it mediates signal transduction (60). Le Bouteiller et al. suggested that CD160 may associate with adaptor proteins containing ITAMs in lipid rafts to initiate its downstream signaling pathway (60). However, Rabot et al. demonstrated that CD160 was unable to associate with DAP10 or DAP12 signaling adaptors (55). In addition, it has been reported that CD160-TM activates NK-cell-activating signaling pathways through its phosphorylated Y225 residue located on its intracellular motif, which can interact with intracellular signaling proteins (57). However, further studies are required to determine how CD160 signaling is mediated. Furthermore, the function of the two truncated isoforms, CD160-ΔIg-GPI and CD160-ΔIg-TM, has not yet been described. Both isoforms have only been detected at the transcriptional level. Therefore, we still do not know whether CD160-ΔIg-GPI and CD160-ΔIg-TM are expressed on the cell surface (57).

Moreover, CD160 is also implicated in the regulation of NK-cell metabolism. It was reported that CD160 expression is positively correlated with the expression of the glucose transporter (GLUT1) and glucose uptake in NK cells, which is indispensable for NK-cell functioning and IFN-γ secretion (14, 78). CD160 expression in NK cells upregulates glucose metabolism through the activation of the AKT/mTOR/s6k signaling pathway (14). In addition, mTORC1 signaling was previously demonstrated to be required for the upregulation of GLUT1 expression and the expression of glycolysis rate-limiting enzymes such hexokinase 2 and lactate dehydrogenase A, which enhance glucose uptake and glycolysis and increase NK-cell functioning (79). Furthermore, CD160 plays an important role in activating the regulatory function of liver-resident CD56^bright^ NK cells against allogenic CD8^+^ T cells (80). Its binding with HLA molecules expressed on activated allogenic T cells activates cytotoxic hepatic NK-cell functions and consequently the killing of activated allogenic T cells. Hence, it has a role in immune tolerance in liver transplantation (80).

Furthermore, NK cells play a crucial role in the antitumor immune response due to their capacity to spontaneously detect and lyse transformed or stressed cells (81). Their function is mediated by different activating receptors, such as NKG2D, CD160, and NKp30, and inhibitory receptors, such as killer cell Ig-like receptors (KIRs) and the CD94/NKG2A heterodimer (82, 83). Synergetic stimulation from a combination of receptors elicits the activation of NK-cell cytotoxicity against tumor cells (84). However, an imbalance of these receptors (that is, upregulation of inhibitory receptors and downregulation of activating receptors) impairs NK-cell function and permits tumor evasion (85, 86). In line with these findings, it was reported that downregulation of CD160 in intrahepatic NK cells induces NK-cell impairment in hepatocellular carcinoma (87). This reduced expression is associated with disease aggressiveness, tumor metastasis, and poorer outcomes (87). In addition, intratumoural CD160^+^ NK cells were found to be more exhausted than peritumoural CD160^+^ NK cells in hepatocellular carcinoma and to produce less IFNγ (87). This exhausted phenotype is due to the expression of TGF-β by tumor cells and inhibits NK-cell functions (87). Similarly, the elevated levels of TGF-β1 in human immunodeficiency virus (HIV)-infected individuals decrease the expression of CD160 in NK cells and enhance HIV disease progression (14). This downregulation may be mediated through the upregulation of specific miRNAs that inhibit CD160 transcription by TGF-β1 (14).

#### 2. 2. 2 CD160 function in endothelial cells

CD160-GPI is expressed in activated endothelial cells in retinal vessels (7). Its expression is elevated in retinal blood vessels of patients with vascular retinal diseases compared with normal individuals (7). CD160-GPI is also increased in endothelial cells of newly formed blood vessels in human colon carcinoma and mouse B16 melanoma but is absent in the vessels of healthy tissues (17). The interaction of CD160 with soluble HLA-G1 induces endothelial cell apoptosis and inhibits fibroblast growth factor 2 (FGF2)- and vascular endothelial growth factor (VEGF)-induced neoangiogenesis *in vivo* (61). Therefore, CD160-GPI has been utilized as a therapeutic target in ocular diseases through the use of anti-CD160-GPI mAbs. These mAbs trigger caspase-dependent apoptosis in endothelial cells and prevent pathological neovascularization (7, 17, 61, 66). Treatment with CL1-R2, an anti-CD160 IgG1 mAb, in a rabbit corneal neovascularization (CNV) model led to significant regression of neovessels (66). Additionally, the use of ELB01101, a humanized anti-CD160 IgG4 mAb, in a monkey model of choroidal neovascularization (ChNV) decreased the number of clinically relevant lesions by 50% and has shown good safety and tolerability (66). Furthermore, the use of CL1-R2 in combination with cyclophosphamide chemotherapy in B16 melanoma-bearing mice decreased size of the tumor vasculature (17). Hence, anti-CD160 mAbs can be used as a potential antiangiogenic treatment in ocular diseases and cancer (17, 61, 66).

#### 2. 2. 3 CD160 function in T cells

Assessments of CD160 function in T cells have produced contradictory results, as CD160 has been reported to both activate and inhibit T cell functions. In previous studies, we demonstrated that CD160 has a coactivating function with the CD3 receptor in a minor subset of CD4^+^ CD160^+^ T cells isolated from inflammatory skin lesions in dermatitis and psoriasis samples (67). This minor subset corresponds to effector memory cytotoxic T lymphocytes characterized by the expression of CD3, CD4, CD160, CD8, CD244, and perforin but lacking CD28 expression (68). In contrast, other studies have demonstrated that CD160 functions as a negative regulator of CD4^+^ T cells and NKT cells (4, 5, 69, 72). CD160 inhibits CD4^+^ T-cell activation and decreases the production of cytokines through its engagement with HVEM (4, 69–71). CD160 receptor acts as a bidirectional switch of T-cell activation since it can produce both positive and negative signals depending on its binding to costimulatory ligands (lymphotoxin-α/LIGHT) or coinhibitory ligands (BTLA/CD160) (4). The inactivation of CD4^+^ T cells is induced by the cooperation of BTLA and CD160 (70). These two proteins bind with the same affinity to cysteine-rich domain 1 (CRD1) of HVEM, with CD160 having a stronger inhibitory signaling function and slower dissociation constant than BTLA (70). CD160-GPI does not contain an ITIM domain. Hence, it does not mediate T-cell inhibition through the phosphorylation of the tyrosine phosphatases SHP-1 or SHP-2 in the same way as BTLA upon its engagement with HVEM (88). However, it was suggested that CD160 induces CD4^+^ T-cell inhibition by modulating binding complexes that translocate to lipid rafts and by reducing the tyrosine phosphorylation of different proteins, such as CD3ζ (4, 70). However, the mechanism underlying CD160-mediated negative signaling is still poorly understood (72). In addition, contradictory reports have indicated that CD160 can activate (9, 89) or inhibit CD8^+^ T-cell functions (65, 90, 91). Nikolova and his colleagues have demonstrated that CD160 acts as a coreceptor in TCR signal transduction in human circulating CD8^+^ T cells (6). It plays a protective role during chronic viral infections by enhancing CD8^+^ T-cell functions (8). It synergizes with TCR signaling to enhance the activation of the PI3K-AKT and MEK-ERK signaling pathways, and it promotes T-cell proliferation and degranulation capacities during HIV infections (8). Similarly, Tan and his colleagues demonstrated that CD160 activates CD8^+^ T-cell effector functions against *Listeria monocytogenes* and allows the secretion of granzyme B, IFN-γ, and TNF-α (9). In contrast, others demonstrated that CD160 interaction with HVEM inhibits CD160^+^ CD8^+^ T cells in patients infected with human T-lymphotropic virus type 1 (HTLV-1), and blockade of this interaction by anti-CD160 mAbs or anti-HVEM mAbs reactivates T-cell function and enhances cytokine production (15). Additionally, CD160^+^ CD8^+^ T cells have lower cytotoxicity in pancreatic cancer patients than CD160^ˉ^ CD8^+^ T cells (65). Additionally, the levels of CD160^+^ CD8^+^ T cells were found to be inversely associated with patient survival. This could be related to the expression of other inhibitory receptors, such as PD-1 and TIM-3, that intrinsically impair the function of T cells in CD160^+^ CD8^+^ T cells (65). Moreover, a recent study showed that CD160 is upregulated in Th1-like cells through IL-23 activity and is implicated in the induction of intestinal inflammation during colitis (11). In addition, *in vivo* studies have demonstrated that recipient animals that receive CD160-deficient Th1-like cells have strong protection against colitis and less inflammation (11). Furthermore, loss of CD160 contributes to the maintenance of a naïve-like phenotype and prevents the differentiation of T cells into an effector phenotype (11). In summary, CD160 mediates cell activation during inflammation and presents an important therapeutic target.

#### 2. 2. 4 Soluble CD160 functions in the regulation of the immune system

Soluble CD160 plays an important role in the regulation of the immune system. It can be released by activated CD56^dim^ CD16^+^ NK cells and mast cells through proteolytic cleavage of membrane-bound CD160-GPI (58, 63). Soluble CD160 regulates adaptive immunity functions, modulates helper T-cell functions and inhibits CD8^+^ T-cell cytotoxicity by preventing the interaction of CD160 with HVEM or MHC-I (58, 63) (**Table 1**). Soluble CD160 also prevents the interaction of the beta subunit of the CD8^+^ T-cell coreceptor with the alpha 3 domain of MHC class I, which inhibits cytotoxic T-cell activity (67). Under pathological conditions, sCD160 is secreted by cancer cells to allow tumor immune escape (18). It was recently reported that only melanoma cells and not normal melanocytes constitutively secrete sCD160 (18). Secreted sCD160 binds to HLA molecules and HVEM on target cells and inhibits the activation of NK-cell cytotoxicity toward their target cells (18).

In addition to its important role in the regulation of different immune cells, CD160 has been implicated in the pathophysiological processes of different diseases, including autoimmune diseases (10), atherosclerosis (12), retinal vascular diseases (7), and chronic viral infections (13). It has also been implicated in the physiopathology of various cancer types, such as melanoma and B-chronic lymphocytic leukemia (CLL) (18, 46).

## 3 CD160 expression and dual function in CLL

CLL is a clonal lymphoproliferative disorder characterized by the proliferation of small, mature-appearing CD5^+^ CD23^+^ B lymphocytes that accumulate in the blood, bone marrow, and secondary lymphoid tissues (21). It is classified into two main subsets depending on the mutational status of the immunoglobulin heavy chain variable region (IGHV) genes of the B-cell receptor (BCR), which influences the prognosis of the disease and predicts overall survival (92, 93). IGHV-mutated CLL (M-CLL) represents the indolent form of the disease and is associated with a favorable prognosis (94). IGHV-unmutated CLL (U-CLL) is an aggressive form of the disease and is associated with a worse prognosis (95).

**Figure 2:**
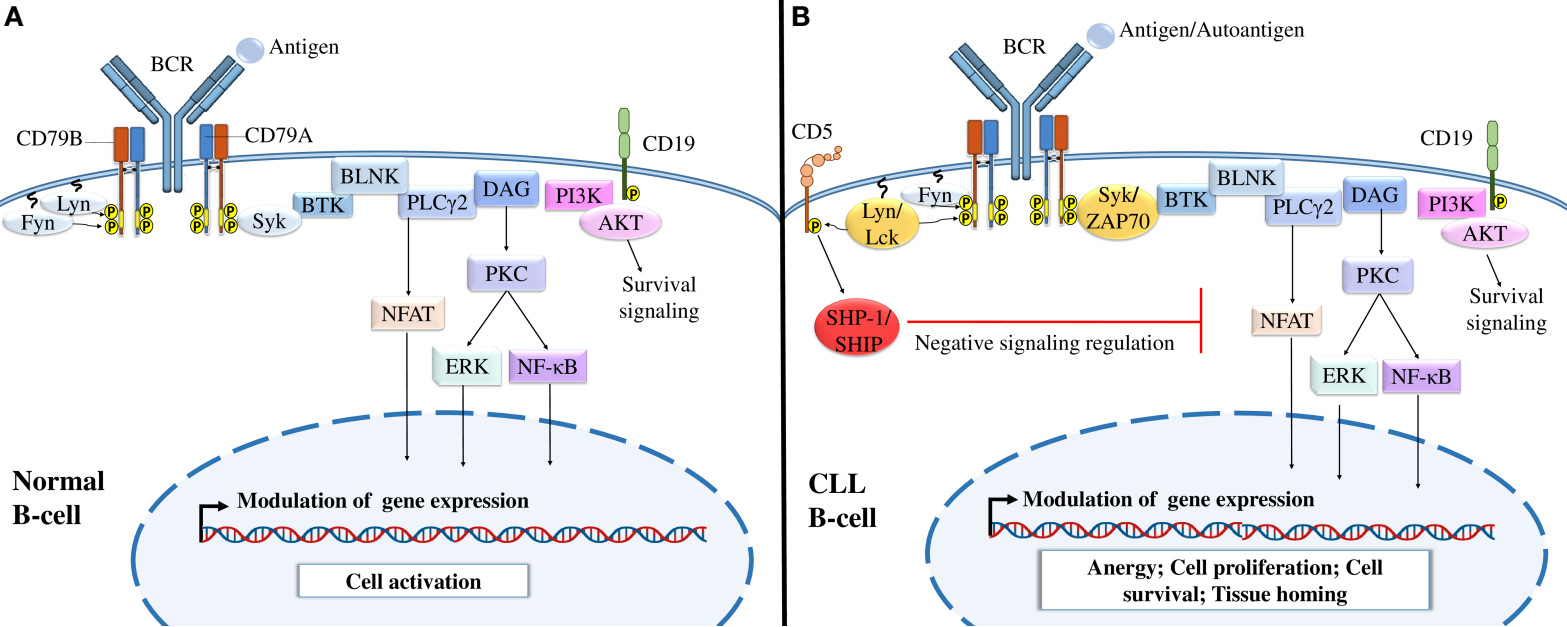
B-cell receptor signaling in normal B cells and CLL cells. **(A)** BCR signaling in normal B cells. The interaction of normal B lymphocyte BCRs with antigens induces the tyrosine phosphorylation of ITAMs by Src-family kinases such as Syk, Fyn, and Lyn, activating different downstream signaling pathways, including the PI3K/Akt, NFAT, NF-κB, and MAPK signaling pathways, which modulate gene expression and activate B cells. **(B)** BCR signaling in CLL B cells. After the interaction of CLL BCRs with antigens or autoantigens, Src-family kinases, such as Lck and ZAP-70 kinases, enhance the activation of different downstream signaling pathways necessary for B-cell survival. Malignant B cells also express CD5, which, upon activation by Lyn kinase recruits SHP-1 or SHIP to dephosphorylate signaling molecules and maintain B-cell anergy. Adapted from (36, 43).

BCR-mediated signaling is a crucial factor in CLL development and CLL cell survival and proliferation (96). Under physiological conditions, BCR targeting activates different signaling pathways that mediate cell activation, proliferation, survival, and migration through the recruitment of kinases, such as lyn, fyn, and syk, to BCR (96) (**Figure 2**). In CLL, malignant B cells express constitutively activated kinases such as Syk and Btk, which induce continuous activation of the cell and upregulation of downstream signaling pathways in the absence of BCR ligands (96–98). In addition to these kinases, CLL cells abnormally express different T-cell markers that are not expressed by normal B cells, such as Lck, Src kinase (a homolog of Lyn) (99), and ZAP70, a protein tyrosine kinase (PTK) associated with the TCR ζ chain (100) (**Figure 2**).

In addition to the prosurvival activity of BCR, CLL cells express other surface markers to survive and proliferate. Phenotypically, CLL cells express different markers of the B lymphocyte lineage, such as CD19, CD23, and CD20 (101, 102). CLL cells are also characterized by low levels of surface IgM expression, which may be a result of a defect in glycosylation and folding of the μIg and CD79a chains and their retention in the endoplasmic reticulum (103). CLL cells also express CD5, a T-cell inhibitory marker (10). CD5 is associated with immunoreceptor tyrosine inhibitory motifs (ITIMs) that, once phosphorylated (104), recruit the tyrosine phosphatases SHP-1 and SHIP, leading to downregulation of BCR-mediated signaling events and continued cell anergy (105).

**Figure 3:**
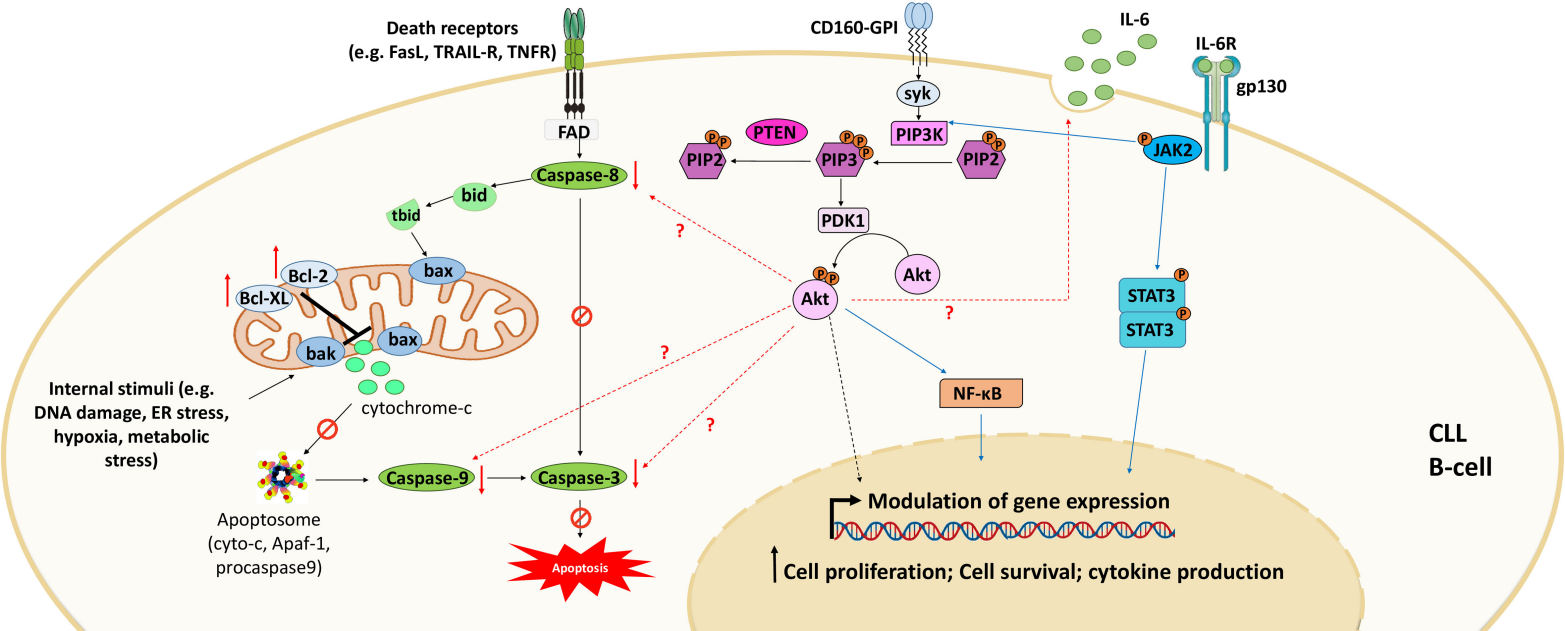
CD160 signaling in CLL. Engagement of CD160-GPI *via* its receptors (MHC-I or HVEM) or monoclonal antibodies leads to activation of the PI3K/Akt signaling pathway to modulate gene expression. This activation also downregulates the expression of pro-apoptotic proteins and upregulates the expression of anti-apoptotic proteins. Consequently, CD160 dysregulates both intrinsic apoptotic pathways (mediated by internal stimuli) and extrinsic apoptotic pathways (triggered by death receptors such as FasR, TRAIL-R, and TNFR). CD160 also activates the expression of cytokines such as IL-6 that activate STAT3 and NF-κB *via* the JAK2/PI3K/Akt axis. Adapted from (46).

CD160 is another surface marker that is aberrantly expressed by CLL cells at all disease stages (19). This marker is normally specific for NK cells and some T-cell subsets (64, 65). It plays a dual role in CLL by triggering both prosurvival and anti-apoptotic signals, which favor cytokine production and cell survival and decrease spontaneous cell death (46) (**Figure 3**). CD160 expression activates prosurvival signaling through the upregulation of PI3K/Akt signaling pathway and increases the secretion of cytokines, mainly the proinflammatory cytokine IL-6 (46); this interleukin activates signal transducer and activator of transcription 3 (STAT3) and NF-κB, which regulate the expression of several genes implicated in CLL cell proliferation and survival (106). CD160 also decreases apoptosis through the downregulation of proapoptotic caspase (caspase-3, -9, and -8) expression and the upregulation of the expression of antiapoptotic proteins, including Bcl-2, Bcl-xL, and Mcl-1, blocking both mitochondria-dependent and mitochondria-independent apoptotic pathways (46, 107). Thus, CD160 prevents apoptosis by blocking cytochrome c release from the outer mitochondrial membrane and inhibits mitochondrial membrane potential decrease and caspase activation (46). However, to understand the complete mechanism underlying CD160-mediated anti-apoptotic effects, further investigation is needed. Additionally, a recent study demonstrated that CD160 hypomethylation in blood cells was correlated with breast cancer in a Chinese population (16). Therefore, further studies should be conducted to demonstrate the mechanisms regulating CD160 expression in both CLL B cells and the neighboring cells that reside in the microenvironment (e.g., T cells and NK cells) and whether this expression is mediated by genetic or epigenetic alterations.

## 4. CD160 expression in the CLL tumor microenvironment

**Figure 4:**
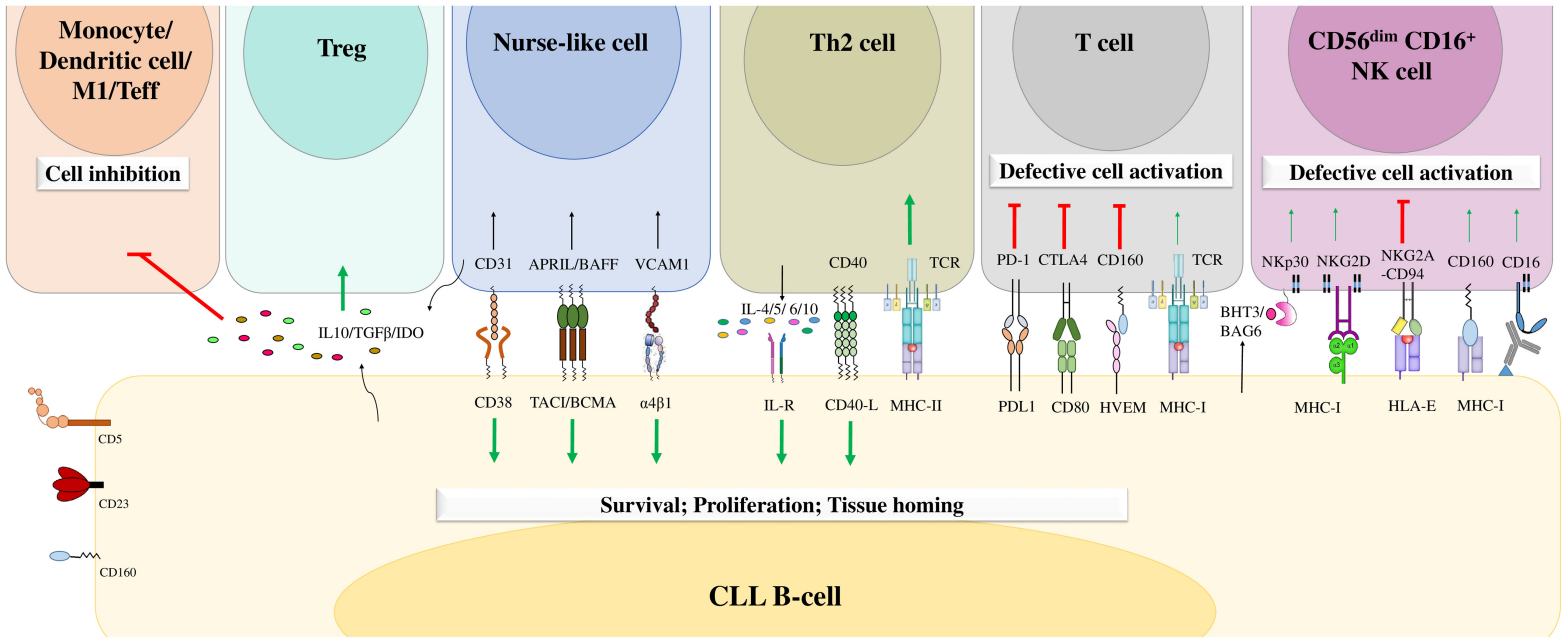
The B-cell chronic lymphocytic leukemia microenvironment. CLL cells engage in crosstalk with different cells in the microenvironment through different molecular interactions or through the release of soluble factors. This crosstalk favors the delivery of activating signals to promote CLL B-cell survival, proliferation, and tissue homing and decreases the numbers of cytotoxic immune cells, leading to tumor cell escape from immune surveillance. Adapted from (36, 113).

CLL cell survival and disease progression are highly influenced by the surrounding microenvironment. It has been reported that tumor B cells are more sensitive to apoptosis when cultured *in vitro* (108). However, they resist apoptosis *in vivo*, which confirms the importance of the microenvironment in CLL cell survival (108). Malignant B cells constantly interact with different cell types in the microenvironment, including T cells (109), mesenchymal stromal cells (32), lymphoma-associated macrophages (110), endothelial cells (29–31), follicular dendritic cells (111), and nurse-like cells (24–28), which negatively or positively influence each other. Furthermore, these neighboring cells in the tumor microenvironment are crucial during all CLL development stages. They can express various chemokines, integrins, cytokines, soluble ligands, adhesion molecules, and survival factors that deliver growth and survival signals to CLL cells, enhance their clonal expansion, and induce drug resistance (36). CLL cells deliver chemokine gradients into lymph nodes, where they form proliferation centers and interact closely with tumor microenvironment components (112). This interaction favors strong activation of BCR-mediated signaling and enhances CLL cell proliferation (112). In addition, CLL cells can also create a favorable microenvironment by regulating the functions of neighboring cells either through the expression of soluble molecules or through direct contact with these cells, inducing their proliferation and survival (110) (**Figure 4**).

Moreover, the antitumor immune response that is mainly mediated by T cells and NK cells is impaired in the CLL microenvironment (114–116). CLL cells overexpress different inhibitory ligands, such as programmed cell death 1 ligand (PD-L1) and HVEM, which bind to PD1 and BTLA, respectively, expressed on cytotoxic T cells and NK cells. These ligands alter T-cell activation, actin polymerization, and immune synapse formation and induce immunosuppression (114–116). This exhaustion phenotype has been described more frequently in T cells located in secondary lymphoid organs than in those circulating in peripheral blood (33). In addition, extracellular vesicles secreted by CLL cells induce phenotypic, functional and transcriptional dysfunction in chimeric antigen receptor (CAR) T cells, leading to their exhaustion (117). Moreover, the exhausted CD8^+^ T cells in patients with CLL express high levels of inhibitory molecules such as CD160, PD-1 and TIGIT (91). This increased expression of inhibitory receptors decreases T-cell proliferation and cytotoxic functions (91). This impairment is also illustrated by the low production of TNF-α and IFN-γ by CD160^+^ CD8^+^ T cells, which are critical cytokines for antitumoral immunity (91). Moreover, the expression of CD160 in CD8^+^ T cells in patients with CLL was found to be correlated with the oversecretion of IL-16 by CLL cells. Hence, Bozorgmehr et al. suggested that IL-16 secretion by CLL cells may have a role in the upregulation of CD160 expression in T cells and the induction of their exhaustion (91). However, further studies should be conducted to confirm these results and analyze the impact of IL-16 on the expression of CD160 in other cell types, such as NK cells.

NK cells remain defective in CLL patients and show impaired production of soluble cytotoxic mediators and reduced degranulation and lysis capacities (113, 118). Under physiological conditions, NK cells express different inhibitory and activating molecules that regulate their own activity and functions (119). However, in CLL, NK-cell exhaustion could result from inhibitory signaling mediated by the engagement of both NKG2A and ILT2 by HLA-E and HLA-G, respectively (120, 121). In addition, other inhibitory receptors are overexpressed in NK cells, including the killer cell Ig-like receptors (KIRs) KIR2DL1 and KIR3DL1 (37), which strengthen the inhibitory signals delivered to these cells. In contrast, NK cells exhibit decreased expression of activating receptors such as CD16, NKG2D, and NKp30, which could contribute to the hyporeactivity of NK cells in CLL (120). However, the expression level of CD160 in NK cells in patients with CLL has not yet been determined. In addition, the expression of CD160 in both NK cells and CLL B cells suggests potential future therapeutic avenues. Future studies should be conducted to evaluate the targeting of CD160 by monoclonal antibodies in CLL, the potential effect of such agents on the reactivation of NK cells, and their induction of antibody-dependent cellular cytotoxicity (ADCC).

## 5 CD160 as a potential prognostic marker for the detection of minimal residual disease in CLL

Minimal residual disease (MRD) is the remaining cancer cells that can be detected after treatment (122). The amount of remaining cells can indicate the efficacy of the treatment and the probability of disease relapse (122). Moreover, the use of targeted therapy as a monotherapy induces partial remission and resistance (123). Therefore, the assessment of MRD is essential for the establishment of an effective therapeutic protocol for CLL and the prevention of relapse, especially after a first-line therapeutic regimen.

MRD assessment in CLL can be performed through the use of three sensitive techniques: real-time polymerase chain reaction (PCR), next-generation sequencing and flow cytometry (124, 125). The European Research Initiative for CLL (ERIC) has developed a gold standard flow cytometry assay that combines 8 antibodies, namely, antibodies against CD5, CD3, CD19, CD20, CD22, CD43, CD79b, and CD81 (126). Novel assays have been developed by combining new prognostic markers such as CD160 into a simple single flow cytometry analysis. The restricted expression of CD160 in CLL and its lack of expression in normal B cells make it a good prognostic marker for the detection of MRD in CLL (54). A single tube flow cytometry assay detecting CD160 (CD160FCA) was developed for the assessment of CLL MRD (54). This assay incorporates six markers, namely, CD160, CD2, CD5, CD19, CD23, and CD45. It allows the quantification of MRD to a level of 10^ˉ4^ (1 malignant cell per 10 000 normal PB cells) in both peripheral blood and bone marrow (54). It also has a high concordance with the gold standard assay (p<0.01). In addition, MRD assessed by CD160FCA after first-line or second-line treatment was found to be correlated with event-free survival (EFS) (54). After first-line treatment, patients with CR and MRD negativity had a prolonged EFS (63 months n=32) compared with patients in CR with MRD positivity (16 months, n=11). Similarly, after second-line treatment, patients with CR and MRD negativity had prolonged EFS (48 months) compared with patients with CR and MRD positivity (24 months) (54).

Another assay combining CD160 and receptor tyrosine kinase-like orphan receptor 1 (ROR1) in a single tube flow cytometry experiment (CD160-ROR1FCA) was developed for the detection of MRD. ROR1 is a tumor-specific antigen of malignant B cells. It is constitutively phosphorylated in CLL and is associated with disease progression (127, 128). CD160-ROR1FCA also includes monoclonal antibodies against CD2, CD5, CD19, CD23, and CD45. The assay showed high sensitivity and had a limit of detection of 0.001%. It also had good correlation (R = 0.98, p < 0.01) with the ERIC gold-standard assay (129). Both assays detect MRD to a level of 10^-5^ (1 malignant cell per 100 000 normal PB cells) (129).

## 6 Conclusions and future directions

Emerging evidence has demonstrated that CD160 participates in CLL pathogenesis by activating PI3K/Akt-independent pathways and inhibiting apoptosis, consequently activating tumor proliferation and inducing resistance to apoptosis. However, further characterization of CD160 signaling pathways in CLL is needed to fully understand its role in CLL pathogenesis. In addition, abnormal expression of CD160 in only CLL cells and its lack of expression in normal B cells make it a potential prognostic marker for the assessment of MRD in CLL. Approaches have been recently developed to assess MRD in CLL through the detection of CD160 and other markers using flow cytometry. These approaches have demonstrated high sensitivity and accuracy, proving the usefulness of CD160 as a prognostic marker. However, the therapeutic use of CD160 in CLL has not been assessed; thus, further related studies should be conducted. Nevertheless, the role of CD160 in CLL pathogenesis and the impairment of antitumor immune cells in the CLL microenvironment makes it a promising target in CLL, and relevant therapeutic approaches may be developed. One potential target that should be assessed is NK cells. These cells are known for their capacity to spontaneously detect and kill tumor cells. Nevertheless, the CLL microenvironment hinders their cytotoxic and antitumor immune functions. Hence, reactivation of exhausted NK cells in the CLL microenvironment is an important therapeutic strategy. One therapeutic approach that could be tested is the use of engineered anti-CD160-GPI mAbs that can engage both CD160-GPI receptors expressed on NK cells and CLL-B cells and FcγRs expressed on NK cells. The engagement of CD160-GPI receptors on NK cells might mediate the reactivation of NK-cell cytotoxicity and the production of cytokines, including IFN-γ and TNF-α. IFN-γ is known for its antitumor activity. It can induce tumor cell cycle arrest and activate the polarization of macrophages into the tumoricidal M1 macrophage subtype (130). It also activates the NADPH-dependent phagocyte oxidase system and upregulates nitric oxide production, contributing to tumor cell death. Moreover, IFN-γ upregulates lysosomal enzymes such as cathepsin B, which enhances cell surface MHC expression on antigen-presenting cells (APCs) and favors the recruitment and activation of effector cells, including CD8 cytotoxic T cells and NK cells (130). IFN-γ also inhibits the differentiation of protumoral immune cells, namely, Treg cells, Th2 cells and Th17 cells (130). Furthermore, IFN-γ can also have direct antitumor activity by activating proapoptotic proteins such as caspase-1, 3, and 8 and upregulating FAS production and the expression of TNF-related apoptosis-inducing ligand (TRAIL) (130). Additionally, the use of anti-CD160 mAbs may also trigger ADCC through the engagement of FcγRs. Moreover, the FcγRs present on phagocytic cells may also recognize anti-CD160 mAbs and activate ADCP against tumor cells. However, this approach may have some limitations. In particular, the use of anti-CD160-GPI mAbs may enhance the proliferation and resistance to apoptosis of CLL B cells rather than activating exhausted NK cells. In addition, the dual function of CD160 in T cells makes it difficult to predict its impact on the activation or inhibition of immune cells in the CLL microenvironment. Hence, proof of concept studies are needed to investigate the effect of anti-CD160 mAbs in the CLL microenvironment.

## Author contributions

LO: conceptualization, manuscript writing, figure artwork. AA: review, editing. AB: conceptualization, review & editing, supervision, project administration; SB: conceptualization, writing - review & editing, supervision, project administration. All authors contributed to the article and approved the submitted version.

## Funding

This work was financially supported by the Mohammed VI Polytechnic University.

## Conflict of interest

The authors declare that the research was conducted in the absence of any commercial or financial relationships that could be construed as a potential conflict of interest.

## Publisher’s note

All claims expressed in this article are solely those of the authors and do not necessarily represent those of their affiliated organizations, or those of the publisher, the editors and the reviewers. Any product that may be evaluated in this article, or claim that may be made by its manufacturer, is not guaranteed or endorsed by the publisher.

## Glossary

**Table:**

ADCC	Antibody-dependent cell cytotoxicity
ADCP	Antibody-dependent cellular phagocytosis
AML-1	Acute myelogenous leukaemia-1
APRIL	A proliferation-inducing ligand
Bcl-2	(B-cell lymphoma 2)
BCMA	B-cell maturation antigen
BCR	B cell receptor
Btk	Bruton’s tyrosine kinase
BTLA	B and T lymphocyte attenuator
CAR	Chimeric antigen receptor
CD160-TM	CD160-transmembrane isoform
CIT	Chemoimmunotherapy
CLBO	Chlorambucil and obinutuzumab
CLL	B-Chronic lymphocytic leukemia
CNV	Corneal neovascularization
CHNV	Choroidal neovascularization
CRD1	Cysteine-rich domain 1
ERK	Extracellular signal-regulated kinase
FCR	Fludarabine cyclophosphamideand rituximab
FGF2	Fibroblast Growth Factor 2
GPI	Glycosylphosphatidylinositol
GLUT1	Glucose transporter 1
HIV	Human immunodeficiency virus
HLA	Human leukocyte antigen
HTLV1	Human T-lymphotropic virus type 1
HVEM	Herpesvirus entry mediator
ICAM	Intercellular Adhesion Molecule
IFN-γ	Interferon-γ
Ig	Immunoglobulin
IGHV	Immunoglobulin heavy chain variable
IL	Interleukin
ITAM	Immunoreceptor tyrosine-based activation motif
ITIM	Immunoreceptor tyrosine-based inhibitory motif
KIR	Killer cell Ig-like receptor
Lck	Lymphocyte-specific protein tyrosine kinase
mAb	Monoclonal antibody
MAPK	Mitogen-activated protein kinase
MHC-I	Major histocompatibility complex
M-IGHV	Mutated immunoglobulin heavy chain variable
mTORC1	Rapamycin complex 1
NFAT	Nuclear factor of activated T-cells
NF-κB	Nuclear factor kappa-light-chain-enhancer of activated B cells
NK	Natural killer
NKG2A	Natural killer group 2A
NKG2D	Natural killer group 2D
NKT	Natural killer T cell
PB	Peripheral blood
PD-1	Programmed cell death 1
PD-L1	Programmed death-ligand 1
PI3K	Phosphoinositide 3-kinase
PLCγ2	Phospholipase Cy2
PTKs	Protein tyrosine kinases
sCD160	Soluble CD160
SFK	Src family kinases
SHP-1	Src homology 2 domain-containing protein tyrosine phosphatase 1
SHIP	Src homology 2 (SH2)-containing inositol phosphatase
STAT	Signal transducer and activator of transcription
Syk	Spleen tyrosine kinase
TNF	Tumor necrosis factor
TRAIL	TNF-related apoptosis-inducing ligand
TSS	Transcription start site
U-IGHV	Unmutated immunoglobulin heavy chain variable
ZAP-70	ζ-associated protein kinase 70
VEGF	Vascular Endothelial Growth Factor
